# Three Heat Shock Protein Genes and Antioxidant Enzymes Protect *Pardosa pseudoannulata* (Araneae: Lycosidae) from High Temperature Stress

**DOI:** 10.3390/ijms232112821

**Published:** 2022-10-24

**Authors:** Di Fu, Jing Liu, Ying-Na Pan, Jia-Yun Zhu, Feng Xiao, Min Liu, Rong Xiao

**Affiliations:** Guizhou Provincial Key Laboratory for Agricultural Pest Management of Mountainous Regions, Institute of Entomology, Guizhou University, Guiyang 550025, China

**Keywords:** *Pardosa pseudoannulata*, heat-shock protein (HSP) genes, antioxidant enzymes, heat stress, RNAi, thermotolerance

## Abstract

*Pardosa pseudoannulata* (*P. pseudoannulata*) is an essential natural predatory enemy in rice ecosystems. The fluctuating climate may cause them to experience heat stress, whereas heat shock proteins (HSPs) and antioxidant enzymes help resist heat damage. Herein, we cloned and characterized the full-length genes *PpHSP27*, *PpHSP60*, and *PpHSC70* from *P. pseudoannulata*. Changes in gene expression levels and superoxide dismutase (SOD), catalase (CAT), and glutathione transferase (GST) activities in adult male and female *P. pseudoannulata* were measured at different stress exposure times and temperatures. We found that the abovementioned HSP genes belong to the sHSP, HSP60, and HSP70 families. The expression of the three HSP genes and the activities of SOD, CAT, and GST were significantly upregulated with the increasing stress temperature and time. The knockdown of the three HSP genes via RNA interference significantly decreased the survival rate of male and female *P. pseudoannulata* during high temperature stress. Thus, *PpHSP27*, *PpHSP60*, and *PpHSC70* play an important role in the heat tolerance of *P. pseudoannulata*, and SOD, CAT, and GST enable recovery heat stress-induced oxidative damage. Their changes and regulation during high temperature stress can improve spiders’ adaptability in the field and enhance the biological control of environmental pests.

## 1. Introduction

Arthropods are relatively susceptible to high temperatures because they do not have mechanisms to regulate their body temperature. High temperatures can significantly affect their growth, development, reproduction, survival, and other processes [1,2]. The intensity, frequency, and duration of high temperatures are increasing because of global warming, and these trends are expected to deteriorate with increased global warming [3,4]. During their long evolution, arthropods have evolved various behavioral, morphological, physiological, and molecular strategies to avoid high-temperature damage. The production of heat shock proteins (HSPs) and the enhancement of antioxidant enzymes are considered important strategies for arthropods to cope with heat stress [5,6,7,8,9].

As with adverse environmental factors, such as hypoxia, heavy metal ion pollution, UV stress, and starvation, heat stress causes protein denaturation and loss of function in the organism [5,8,10]. As a chaperone protein, HSP cooperates with auxiliary molecular chaperones and auxiliary proteins to mediate activities such as protein folding, localization, and degradation, and prevents the protein denaturation caused by the abovementioned reasons [11,12,13]. Based on the relative molecular weight and amino acid (AA) sequence homology, HSPs may be divided into HSP110, HSP90, HSP70, HSP60, small heat shock proteins (sHSPs), and other families [13,14,15]. Of these, HSP70 is the most phylogenetically conserved and widely studied HSP in arthropods [9,16,17]. In arthropods, HSPs play a protective role in various tissues and organs. For example, HSPs can protect the normal neural circuit function of *Drosophila* under heat stress [18]. HSP genes in the midgut of *Rhodnius prolixus* and *Aedes aegypti* were significantly increased following the consumption of a hot blood meal, suggesting that HSP genes protect their midgut from the heat stress caused by hot blood [19,20]. Within the same species, there are also differences in the expression of HSP genes. In *Nilaparvata lugens*, there is a difference in heat tolerance between the two morphs: the macropters with migratory ability are more heat tolerant than the brachypters, and RNA interference (RNAi) experiments showed that this difference results from the overexpression of HSP genes [21]. Welte et al. [22] found that *Drosophila melanogaster* had the highest *HSP70* genes expression in the first instar larvae, which were more thermotolerant than other instars, and the induction of *HSP70* genes enhanced the heat tolerance of *D. melanogaster* in the first instar. The expression levels of the *HSP70* and *HSP23* genes were significantly higher in *Bemisia tabaci* females than in males. After knocking down these genes, heat tolerance was significantly decreased in females, whereas no change was observed in males. This means that the high expression of HSP genes in females results in a higher survival rate under conditions of heat stress [23]. These findings suggest that HSP genes play an important role in heat stress resistance in different tissues and at various developmental stages in arthropods.

Reactive oxygen species (ROS) are byproducts of metabolism in the mitochondria, endoplasmic reticulum, and lysosomes [24]. Low levels of ROS favor cell signaling and the induction of defense genes in insects, but if overproduced, it can result in oxidative damage to lipids, proteins, and nucleic acids within cells [9,25]. To cope with this damage, heat stressed arthropods reduce cell damage through antioxidant enzyme [7,26]. The main antioxidant enzymes in arthropods are superoxide dismutase (SOD), catalase (CAT), and glutathione transferase (GST) [26,27,28,29,30]. The function of SOD is to degrade superoxide anions (O^2−^) to hydrogen peroxide (H_2_O_2_) and oxygen (O_2_), following which CAT decomposes H_2_O_2_ to H_2_O and O_2_. GST not only catalyzes the reduction of H_2_O_2_ but also that of lipid H_2_O_2_ [31,32,33,34].

Rice field spiders are important natural enemies of rice field pests [35,36]. *Pardosa pseudoannulata* is one of the dominant species of rice field spiders, which is widely distributed across Asia [37]. It has the characteristics of high activity and strong predatory ability, fecundity, and resistance [35]. Environmental temperature is one of the most critical factors affecting the development, survival, reproduction, and predation of *P. pseudoannulata* [38]. The optimal temperature range for the survival of *P. pseudoannulata* is 20–32 °C. At a temperature of 25 °C, spawning amounts and egg hatching rates are the highest; however, when the temperature exceeds 35 °C, the lifespan of *P. pseudoannulata* is significantly shortened [35,39,40]. In paddy fields in southern China, the summer temperature often exceeds 40 °C [41]. Therefore, *P. pseudoannulata* in the paddy field system is often subjected to high temperature stress; thus, it is crucial to understand the molecular and physiological mechanisms of *P. pseudoannulata* in the context of high temperature resistance.

The functional studies of HSP genes and antioxidant enzymes in heat stress have made important progress in other arthropods, but the underlying mechanism of action in spiders requires further elucidation. In a previous study by our group, we performed a transcriptome sequencing study of *P. pseudoannulata* and found that the *HSP27*, *HSP60* and *HSC70* genes were significantly upregulated after high temperature stress, suggesting that they may have an essential role in the *P. pseudoannulata*’s resistance to high temperature stress [42]. Therefore, this study aimed to measure the changes in antioxidant enzyme activities and *PpHSP27*, *PpHSP60*, and *PpHSC70* gene expression levels in male and female *P. pseudoannulata* after exposure to various temperatures (31 °C, 34 °C, 37 °C, 40 °C, and 43 °C) and for different durations (1, 2, 4, 8, and 12 h). In addition, changes in high temperature tolerance mechanisms of male and female adult *P. pseudoannulata* were observed after silencing the three abovementioned HSP genes. Our findings can provide insight into the molecular and physiological mechanisms of spider resistance to high temperatures, as well as a theoretical basis for spider protection and utilization.

## 2. Results

### 2.1. Sequence Analysis and Phylogenetic Tree

**Figure 1:**
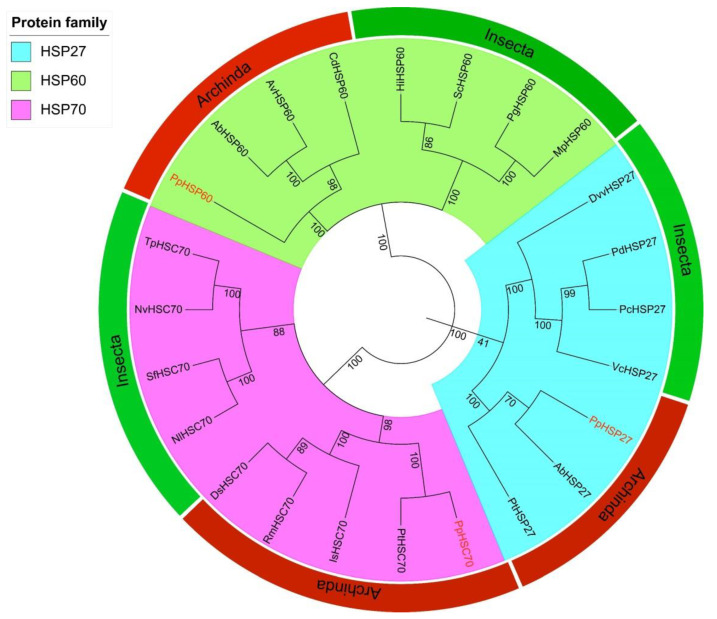
The phylogenetic tree constructed from the deduced amino acid (AA) sequences of *HSP27*, *HSP60*, and *HSP70* in *P. pseudoannulata*, Archinda and Insecta. Numbers at the nodes are bootstrap values (1000 replications). The organisms and accession numbers were as follows: DvvHSP27 = *Diabrotica virgifera virgifera* (XP028134524.1), PdHSP27 = *Polistes dominula* (XP015186657.1), PcHSP27 = *Polistes canadensis* (XP014612919.1), VcHSP27 = *Venturia canescens* (XP043268524.1), PpHSP27 = *Pardosa pseudoannulata* (ON808409), AbHSP27 = *Argiope bruennichi* (KAF8778562.1), PtHSP27 = *Parasteatoda tepidariorum* (XP015921832.1),PpHSP60 = *P. pseudoannulata* (ON808407), AbHSP60 = *A. bruennichi* (KAF8796449.1), AvHSP60 = *Araneus ventricosus* (GBN34335.1), CdHSP60 = *Caerostris darwini* (GIX94638.1), HiHSP60 = *Hermetia illucens* (XP037915503.1), ScHSP60 = *Stomoxys calcitrans* (XP013103765.1), PgHSP60 = *Pseudomyrmex gracilis* (XP020293187.1), MpHSP60 = *Monomorium pharaonis* (XP012536851.1),TpHSC70 = *Trichogramma pretiosum* (XP014221706.1), NvHSC70 = *Nasonia vitripennis* (XP001599525.2), SfHSC70 = *Sogatella furcifera* (QIQ19556.1), NlHSC70 = *Nilaparvata lugens* (AQP31367.1), DsHSC70 = *Dermacentor silvarum* (XP037555227.1), RmHSC70 = *Rhipicephalus microplus* (XP037283294.1), IsHSC70 = *Ixodes scapularis* (XP029836651.2), PtHSC70 = *P. tepidariorum* (XP042896938.1), PpHSC70 = *P. pseudoannulata* (ON808408).

**Figure 2:**
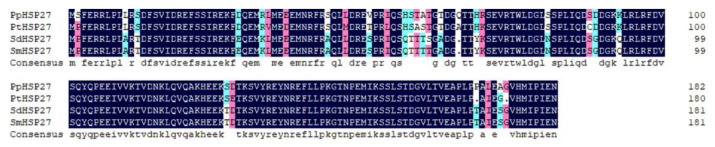
Multiple alignment of the deduced AA sequence of *PpHSP27* with that of other species. Pt, *P. tepidariorum* (XP015921832.1); Sd, *Stegodyphus dumicola* (XP035231886.1); *Sm*, *Stegodyphus mimosarum* (KFM65662.1).

The full-length sequences of the three HSP genes *PpHSP27*, *PpHSP60*, and *PpHSC70* were obtained using RT-PCR and RACE technology. The full length of *PpHSP27* (GenBank Accession No. ON808409) was 997 base pairs (bp), included a 5′ untranslated region (UTR) of 108 bp, a 3′ UTR of 337 bp contained a typical polyA tail, and an open reading frame (ORF) of 552 bp. The ORF encoded 183 AAs and its predicted molecular weight was 21.18 kDa. The theoretical isoelectric point (pI) is 5.50. The instability index (II) was 55.25, indicating that it is an unstable protein. The grand average of hydropathicity (GRAVY) was −0.763, indicating that it is a hydrophilic protein. The deduced AA sequence of *PpHSP27* contained an alpha crystallin domain (ACD), which is a structural feature of the sHSP family (Figure S1). In the phylogenetic tree, HSP27 was divided into two taxa, Arachnida and Insecta, and *P. pseudoannulata* was clustered with Arachnida (Figure 1). The deduced AA sequences were all greater than 88% similar to HSP27 from *Parasteatoda tepidariorum* (XP015921832.1), *Stegodyphus dumicola* (XP035231886.1), and *Stegodyphus mimosarum* (KFM65662.1) (Figure 2).

**Figure 3:**
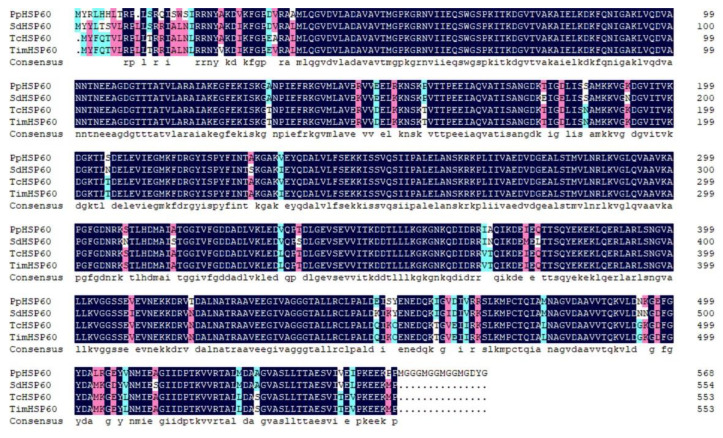
Multiple alignment of the deduced AA sequence of *PpHSP60* with that of other species. Sd, *S. dumicola* XP035231886.1; Tc, *Trichonephila clavata* GFR30939.1; Tim, *Trichonephila inaurata madagascariensis* GFS44331.1.

The full length of *PpHSP60* (GenBank Accession No. ON808407) was 2065 bp, included a 5′ UTR of 158 bp, a 3′ UTR of 199 bp contained a typical polyA tail, and an ORF of 1710 bp. The ORF encodes 569 AAs and its predicted molecular weight was 61.17 kDa. The pI was 5.43. The II was 31.6, which indicated that it is a stable protein. The GRAVY was −0.184, which indicated that it is a hydrophilic protein. The deduced AA sequence of *PpHSP60* contained a characteristic sequence of the HSP60 family, AAVEEGIVAGGG (Figure S2). In the phylogenetic tree, HSP60 was divided into two taxa, Arachnida and Insecta, and *P. pseudoannulata* was clustered with Arachnida (Figure 1). The deduced AA sequences were all greater than 92% similar to HSP60 from *S. dumicola* (XP035227740.1), *Trichonephila clavata* (GFR30939.1), and *Trichonephila inaurata madagascariensis* (GFS44331.1) (Figure 3).

**Figure 4:**
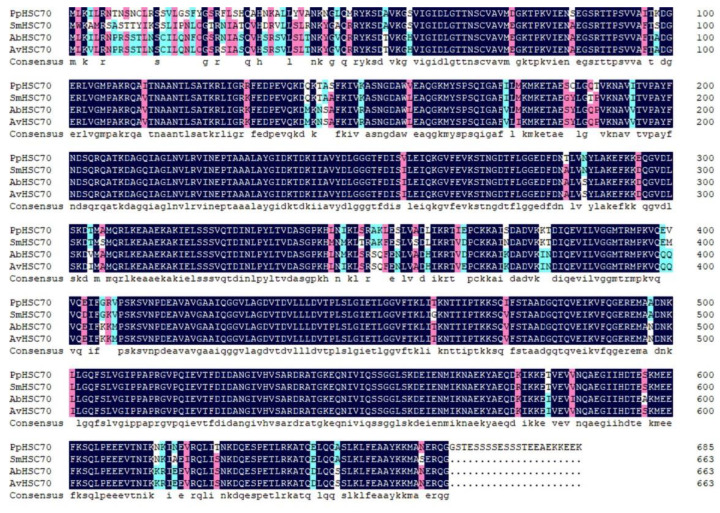
Multiple alignment of the deduced AA sequence of *PpHSC70* with that of other species. Sm, *S. mimosarum* KFM81131.1; Ab, *A. bruennichi* KAF8774081.1; Av, *A. ventricosus* GBM18724.1.

The full length of *PpHSC70* (GenBank Accession No. ON808408) was 2438 bp, including a 5′ UTR of 134 bp, a 3′ UTR of 247 bp containing a typical polyA tail, and an ORF of 2061 bp. Its ORF encodes 686 AAs and its predicted molecular weight was 74.56 kDa. The pI was 6.19. The II was 41.78, which indicated that it is a stable protein. The GRAVY was −0.369, which indicated that it is a hydrophilic protein. The deduced AA sequence of *PpHSC70* contained three HSP70 family signature sequences: IDLGTTNS, VYDLGGGTFDISVL, and VILVGGMTRMPKVQE (Figure S3). In the phylogenetic tree, HSC60 was divided into two taxa, Arachnida and Insecta, and *P. pseudoannulata* was clustered with Arachnida (Figure 1). The deduced AA sequences were all more than 89% similar to HSC70 from *S. mimosarum* (KFM81131.1), *Argiope bruennichi* (KAF8774081.1), and *Araneus ventricosus* (GBM18724.1) (Figure 4).

### 2.2. Effects of High Temperature Stress on PpHSP Genes Expression

**Figure 5:**
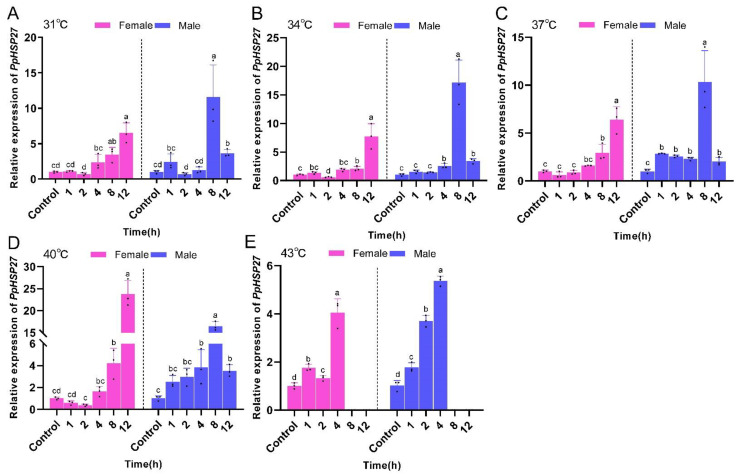
Relative mRNA expression of *PpHSP27* in *P. pseudoannulata* after high temperature stress. (**A**) 31 °C treatment group, (**B**) 34 °C treatment group, (**C**) 37 °C treatment group, (**D**) 40 °C treatment group, (**E**) 43 °C treatment group. Each value represents the mean ± standard deviation (SD) of three replications (*n* = 3). Separate one-way Analysis of Variances (ANOVAs) were used for each temperature and gender, followed by pairwise comparisons at different time points using Tukey’s Honest Significant Difference (HSD) method with different lowercase letters indicating significant differences in pairwise comparisons (*P* < 0.05). No data were available at 43 °C of 8 h and 12 h exposure due to the death of tested spiders.

The expression of *PpHSP27*, *PpHSP60*, and *PpHSC70* in *P. pseudoannulata* were significantly affected by the temperature and time of heat stress as well as their interactions. At the same temperature, the expression of *PpHSP27* in female *P. pseudoannulata* exhibited an overall increasing trend with increasing stress time. The highest expression was observed in the treatment group with the longest stress duration. The expression of *PpHSP27* was not significantly different from that of the control after 1 or 2 h of stress at 31, 34, 37, and 40 °C. The trend of *PpHSP27* expression in male *P. pseudoannulata* was consistent with that in females, and overall, its expression was highest after 8 h of stress. For the same duration of stress, the expression of the *PpHSP27* gene in female *P. pseudoannulata* was highest at the highest stress temperature. The expression of *PpHSP27* in female *P. pseudoannulata* was highest after 12 h of stress at 40 °C and was 25.75-fold (*F* = 93.615, *df* = 17, *P* < 0.001) higher than that of the control. *PpHSP27* expression in male *P. pseudoannulata* was highest after 8 h of stress at 34 °C and 17.14-fold (*F* = 97.336, *df* = 17, *P* < 0.001) higher than that of the control, both significantly higher than their respective controls (Figure 5).

**Figure 6:**
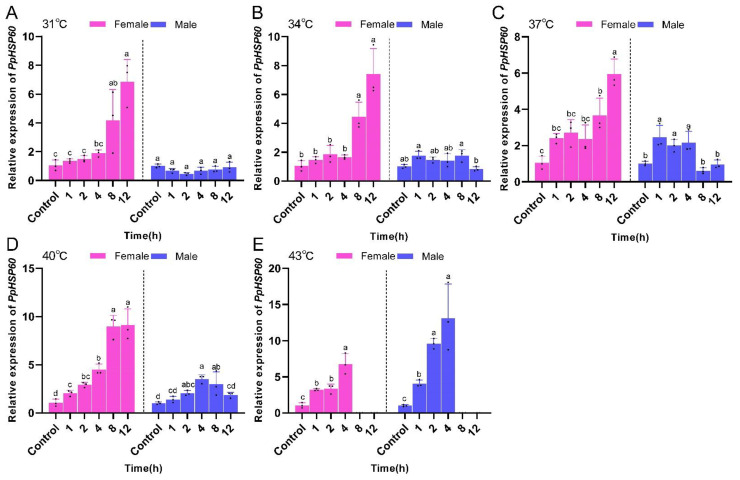
Relative mRNA expression of *PpHSP60* in *P. pseudoannulata* after high temperature stress. (**A**) 31 °C treatment group, (**B**) 34 °C treatment group, (**C**) 37 °C treatment group, (**D**) 40 °C treatment group, (**E**) 43 °C treatment group. Each value represents the mean ± SD of three replications (*n* = 3). Separate one-way ANOVAs were used for each group of temperature or gender, followed by pairwise comparisons at different time points using Tukey’s HSD method with different lowercase letters indicating significant differences in pairwise comparisons (*P* < 0.05). No data were available at 43 °C of 8 h and 12 h exposure due to the death of tested spiders.

Under the same temperature stress, *PpHSP60* gene expression in female *P. pseudoannulata* was highest in all groups, with the longest duration of stress treatment and overall expression increasing with time. The expression of the *PpHSP60* gene in male *P. pseudoannulata* showed a general trend of increasing and then decreasing with time. The expression of *PpHSP60* was not significantly different from the control group under the five time periods of stress at 31 °C and 34 °C. At the same duration of stress, *PpHSP60* expression in both male and female *P. pseudoannulata* exhibited the highest expression in the highest temperature treatment group. *PpHSP60* expression was highest in female *P. pseudoannulata* after 12 h of stress at 40 °C, which was 9.12-fold (*F* = 59.169, *df* = 17, *P* < 0.001) higher than the control. In males after 4 h of stress at 43 °C, the expression was 13.14-fold (*F* = 92.728, *df* = 11, *P* < 0.001) higher than the control, and both were significantly higher than their respective controls (Figure 6).

**Figure 7:**
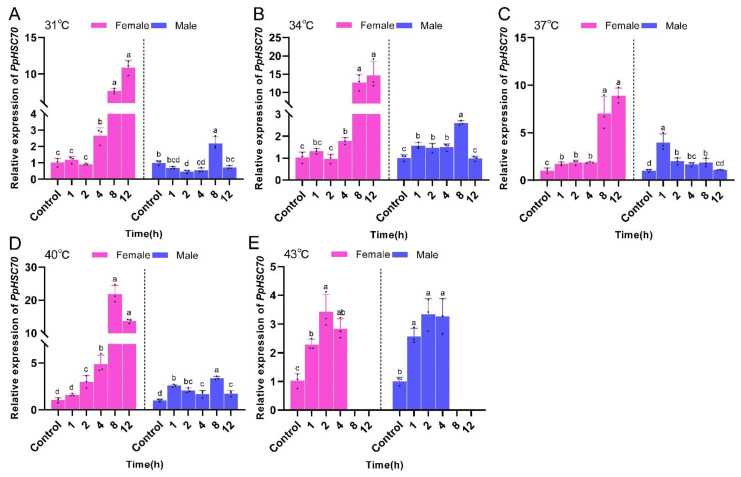
Relative mRNA expression of *PpHSC70* in *P. pseudoannulata* after high temperature stress. (**A**) 31 °C treatment group, (**B**) 34 °C treatment group, (**C**) 37 °C treatment group, (**D**) 40 °C treatment group, (**E**) 43 °C treatment group. Each value represents the mean ± SD of three replications (*n* = 3). Separate one-way ANOVAs were used for each group of temperature or gender, followed by pairwise comparisons at different time points using Tukey’s HSD method with different lowercase letters indicating significant differences in pairwise comparisons (*P* < 0.05). No data were available at 43 °C of 8 h and 12 h exposure due to the death of tested spiders.

At the same temperature, the expression of the *PpHSC70* gene in female *P. pseudoannulata* exhibited an overall increasing trend with time and the expression of the *PpHSC70* gene in male *P. pseudoannulata* increased and then decreased with time. *PpHSC70* expression was highest in female *P. pseudoannulata* after 8 h of stress at 40 °C, which was 21.85-fold (*F* = 146.728, *df* = 17, *P* < 0.001) higher than the control. In males after 1 h of stress at 37 °C, the expression was 3.97-fold (*F* = 24.665, *df* = 17, *P* < 0.001) higher than the control, and both were significantly higher than their respective controls (Figure 7).

### 2.3. Changes in Antioxidant Enzyme Activity

**Figure 8:**
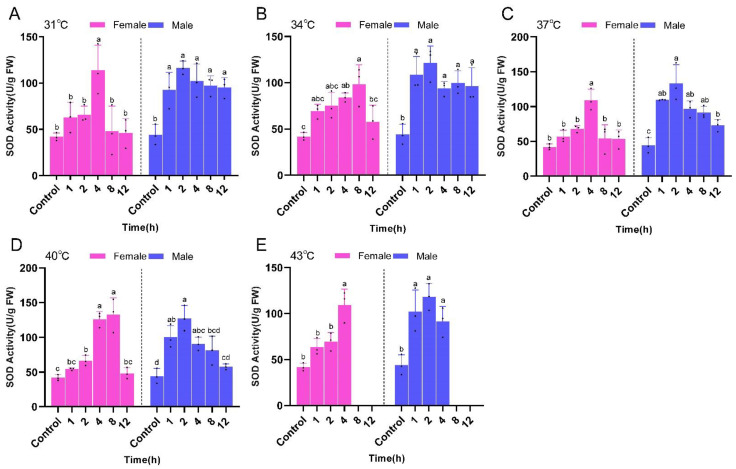
Superoxide dismutase (SOD) activity in *P. pseudoannulata* after high temperature stress. (**A**) 31 °C treatment group, (**B**) 34 °C treatment group, (**C**) 37 °C treatment group, (**D**) 40 °C treatment group, (**E**) 43 °C treatment group. Each value represents the mean ± SD of three replications (*n* = 3). Separate one-way ANOVAs were used for each group of temperature or gender, followed by pairwise comparisons at different time points using Tukey’s HSD method with different lowercase letters indicating significant differences in pairwise comparisons (*P* < 0.05). No data were available at 43 °C of 8 h and 12 h exposure due to the death of tested spiders.

The activities of SOD, CAT, and GST in *P. pseudoannulata* were significantly altered by the temperature and time of heat stress and their interactions. At the same temperature, SOD activities in both male and female *P. pseudoannulata* exhibited a trend of increasing and then decreasing with increased stress time. The SOD activity of female *P. pseudoannulata* was not significantly different from that of the control after 12 h of stress at different temperatures. SOD activity of the male *P. pseudoannulata* in all temperature treatment groups was highest after 2 h of stress and was significantly higher than that of the control group. The highest SOD activity occurred after 8 h of stress at 40 °C for female *P. pseudoannulata* (132.78 U/g FW, *F* =46.588, *df* = 17, *P* < 0.001) and 2 h of stress at 37 °C for males (133.01 U/g FW, *F* = 19.421, *df* = 17, *P* < 0.001). Both were significantly higher than their respective controls (Figure 8).

**Figure 9:**
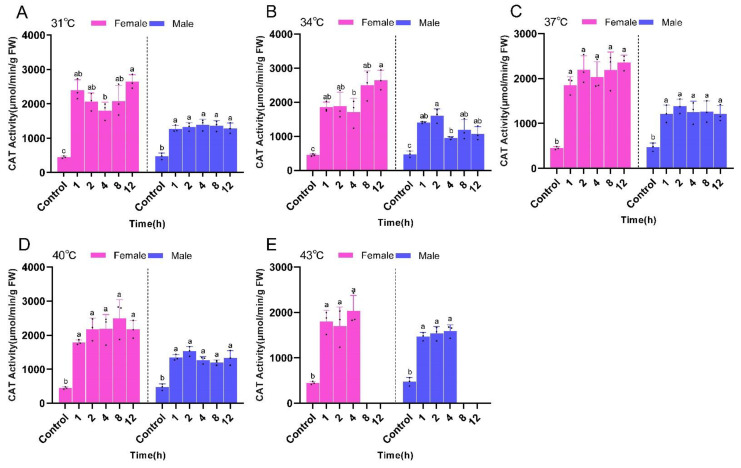
Catalase (CAT) activity in *P. pseudoannulata* after high temperature stress. (**A**) 31 °C treatment group, (**B**) 34 °C treatment group, (**C**) 37 °C treatment group, (**D**) 40 °C treatment group, (**E**) 43 °C treatment group. Each value represents the mean ± SD of three replications (*n* = 3). Separate one-way ANOVAs were used for each group of temperature or gender, followed by pairwise comparisons at different time points using Tukey’s HSD method with different lowercase letters indicating significant differences in pairwise comparisons (*P* < 0.05). No data were available at 43 °C of 8 h and 12 h exposure due to the death of tested spiders.

Overall, CAT activity in female *P. pseudoannulata* was significantly higher than that of the control group after different times of stress at the same temperature, and did not differ significantly from each other within the same temperature treatment group as did the male *P. pseudoannulata*. The highest CAT activity occurred after 12 h of stress at 34 °C for female *P. pseudoannulata* (2647.51 μmol/min/g FW, *F* = 17.338, *df* = 17, *P* < 0.001) and after 2 h of stress at 34 °C for the males (1603.44 μmol/min/g FW, *F* = 21.592, *df* = 17, *P* < 0.001). Both were significantly higher than their respective controls (Figure 9).

**Figure 10:**
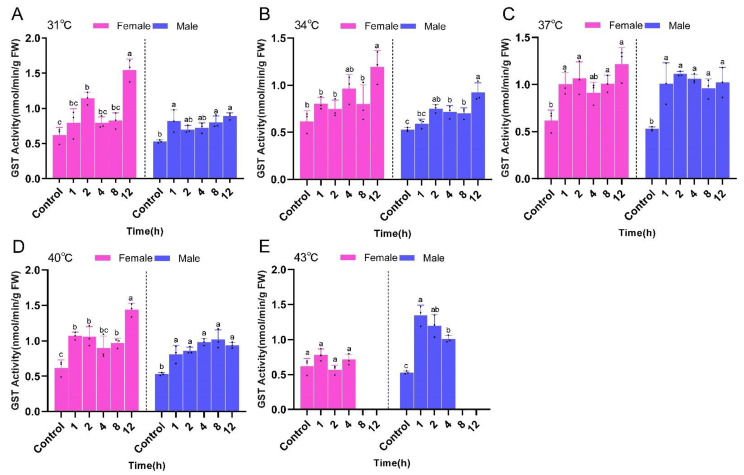
Glutathione transferase (GST) activity in *P. pseudoannulata* after high temperature stress. (**A**) 31 °C treatment group, (**B**) 34 °C treatment group, (**C**) 37 °C treatment group, (**D**) 40 °C treatment group, (**E**) 43 °C treatment group. Each value represents the mean ± SD of three replications (*n* = 3). Separate one-way ANOVAs were used for each group of temperature or gender, followed by pairwise comparisons at different time points using Tukey’s HSD method with different lowercase letters indicating significant differences in pairwise comparisons (*P* < 0.05). No data were available at 43 °C of 8 h and 12 h exposure due to the death of tested spiders.

GST activities of both male and female *P. pseudoannulata* increased with increasing stress time at the same temperature and was significantly higher than that of the control group. However, there was no significant difference between the treatment groups at the same temperature. The highest GST activity appeared after 12 h of stress at 31 °C for female *P. pseudoannulata* (1.55 nmol/min/g FW, *F* = 19.180, *df* = 17, *P* < 0.001) and after 1 h of stress at 43 °C for the males (1.35 nmol/min/g FW, *F* = 37.571, *df* = 17, *P* < 0.001)). Both were significantly higher than their respective controls (Figure 10).

### 2.4. Effect of Silencing Three HSP Genes on High Temperature Tolerance in P. pseudoannulata

**Figure 11:**
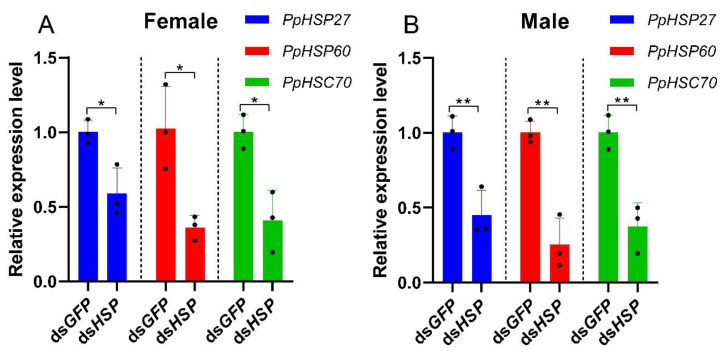
Transcription levels of the *PpHSP27*, *PpHSP60*, and *PpHSC70* genes after silencing. Each value represents the mean ± SD of three replications (*n* = 3). The * means significant differences between ds*GFP* and ds*HSP* according to a Student’s *t*-test. (* *P* < 0.05 and ** *P* < 0.01).

To further understand the role of the three HSP genes in heat resistance of *P. pseudoannulata*, we injected dsRNA into male and female *P. pseudoannulata* and examined changes in the survival rate of male and female *P. pseudoannulata* after high temperature stress. When compared with the control group injected with green fluorescent protein (GFP, GenBank accession number: L29345.1), the expression of the three HSP genes in female *P. pseudoannulata* injected with ds*PpHSP27*, ds*PpHSP60*, and ds*PpHSC70* decreased by 41% (*t* = 3.775, *df* = 4, *P* = 0.020), 64% (*t* = 3.913, *df* = 4, *P* = 0.017), and 59% (*t* = 4.450, *df* = 4, *P* = 0.011), respectively. The expression levels of the three HSP genes in male *P. pseudoannulata* decreased by 55% (*t* = 4.864, *df* = 4, *P* = 0.008), 75% (*t* = 6.674, *df* = 4, *P* = 0.003), and 63% (*t* = 5.571, *df* = 4, *P* = 0.005), respectively. After interference, the expression levels of the three genes in male and female *P. pseudoannulata* were significantly lower than that in the control group (Figure 11).

**Figure 12:**
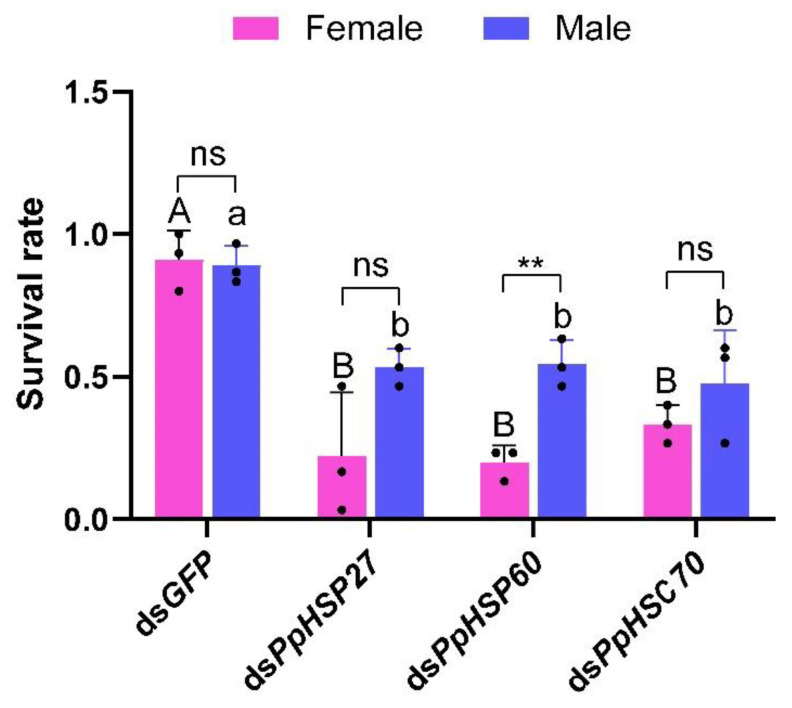
Effects of silencing *PpHSP27*, *PpHSP60*, and *PpHSC70* genes on *P. pseudoannulata*. Each value represents the mean ± SD of three replications (*n* = 3). Separate one-way ANOVAs were used for two genders, followed by pairwise comparisons with different dsRNA processing using Tukey’s HSD method, and different capital letters indicate significant differences among females and different lowercase letters indicate significant differences among males (*P* < 0.05). The * means significant differences between females and males based on a Student’s *t*-test. (* *P* < 0.05 and ** *P* < 0.01).

After injection of ds*PpHSP27*, ds*PpHSP60*, and ds*PpHSC70*, the survival rates of male and female *P. pseudoannulata* were significantly lower than that of the control group injected with ds*GFP* after exposure to high temperature stress at 43 °C for 4 h. After 4 h of high temperature stress at 43 °C, the survival rates of female *P. pseudoannulata* injected with ds*PpHSP27*, ds*PpHSP60*, and ds*PpHSC70* were 22%, 20%, and 33%, respectively, which was significantly lower than the 91% of the control group (*F* = 19.655, *df* = 11, *P* < 0.05). The survival rates of male *P. pseudoannulata* were 53%, 54%, and 48%, respectively, significantly higher than 89% in the control group (*F* = 8.575, *df* = 11, *P* < 0.05) (Figure 12). These results indicated that knockdown of the three HSP genes can significantly reduce the heat tolerance of *P. pseudoannulata* to high temperatures.

## 3. Discussion

High temperature is one of the main factors that affect *P. pseudoannulata*, which leads to decreased predation and a shortened lifespan [39,43]. This may indirectly lead to increased pests in rice fields and raise the cost of people to control pests. It is well-established that HSPs and antioxidant enzymes play an important role in arthropod resistance to high temperature stress. Previous studies suggested that HSPs and antioxidant enzymes may be key factors in the resistance of *P. pseudoannulata* to high temperature stress [44,45]. HSPs are highly conserved in arthropods [5]. In the present study, we cloned and characterized the full-length *PpHSP27*, *PpHSP60*, and *PpHSC70* genes of *P. pseudoannulata,* which will improve the HSP gene data of *P. pseudoannulata* and provide a reference for future studies. The deduced AA sequences show that they contain characteristic sequences of HSP27, HSP60, and HSP70 families and the AA sequence alignment shows that they are highly similar to the HSP27, HSP60, and HSC70 of other Arachnids. Based on our analysis, the three HSP genes isolated from *P. pseudoannulata* belonged to the HSP27, HSP60, and HSP70 families.

There are sexual dimorphisms in *P. pseudoannulata* and there is a huge difference in body size between males and females, thus it is necessary to study male and female *P. pseudoannulata* separately. Our study showed that the expression of the *PpHSP27*, *PpHSP60*, and *PpHSC70* genes increased significantly with increasing stress temperature and stress duration in all treatment groups. This is consistent with the results of *Agasicles hygrophila*, *Neoseiulus barkeri*, and *Scylla paramamosain* [46,47,48]. From this, we hypothesized that these three HSP genes may help *P. pseudoannulata* to resist heat stress. The *HSC70* gene is considered to be constitutively expressed under non-stress conditions and heat stress does not readily affect its expression [49,50]. However, in recent studies on *Tetranychus urticae*, *Sitodiplosis mosellana*, *Pteromalus puparum*, *Plutella xylostella*, as well as others, the expression of the *HSC70* gene increased significantly with increasing temperature [51,52,53,54]. In the present study, we also found that *PpHSC70* gene expression in *P. pseudoannulata* was induced by high temperatures and significantly different from that of the control. This suggested that *HSC70* and *HSP70* have the same function in *P. pseudoannulata*. On average, the expression levels of the three HSP genes in *P. pseudoannulata* were not significantly different from those in the control group after stress at 31 °C and 34 °C for 1 and 2 h, which indicated that the results of short-term stress below 34 °C is not a harsh condition for *P. pseudoannulata*. This was consistent with the results of Zhao et al. [40], who found that 25–32 °C is the optimal temperature for *P. pseudoannulata* to survive. Although the synthesis of HSPs improves the body’s resistance, it also consumes the organism’s energy [55,56]. Thus, for *P. pseudoannulata*, energy may be used in more important places, such as spawning and reproduction, when the external conditions are suitable for survival. Multiple studies have shown that females are more tolerant than males to heat stress, for example, this phenomenon is associated with higher expression of the HSP genes in *B. tabaci* females [23,57,58,59]. In this study, we found that with the increase in stress time, the expression of three HSP genes in *P. pseudoannulata* continued to increase, and although the expression of three HSP genes in male spiders also increased, they decreased significantly after 8 h. Females *P. pseudoannulata* generally play a significant role in reproduction and are larger than males, and more energy is needed to store in their bodies for reproduction, which means they have more energy to regulate themselves against unfavorable conditions when they are exposed to unfavorable conditions [45]. Some studies showed that females were more resistant than males and survived longer than males without water or food [60]. In summary, we speculate that female *P. pseudoannulata* have a higher tolerance than male *P. pseudoannulata* over prolonged high temperature stress.

High temperature stress leads to excessive ROS production in arthropod cells, which in turn, causes damage to the arthropods themselves [9,25]. To avoid such adverse effects, arthropods have evolved the ability to maintain a balanced relationship between ROS and antioxidants [61]. In the present study, the SOD, CAT, and GST activities of *P. pseudoannulata* were significantly increased by stress temperature as well as stress time, suggesting that these enzymes are involved in the scavenging of excess ROS in *P. pseudoannulata*. Studies of *Ophraella communa*, *Corythucha ciliate*, *Mononychellus mcgregori*, and *Hylyphantes graminicola* also confirmed this result [62,63,64,65]. Overall, the SOD activities of male and female *P. pseudoannulata* were not significantly different from that of the control group after 12 h of heat stress at 37 °C and 40 °C. A similar phenomenon was reported for SOD activity in *S. paramamosain* [48]. Based on these results, we hypothesized that there is a limit to the regulatory capacity of SOD and when stress conditions exceed a certain level, the spider may scavenge the generated ROS through other mechanisms. The CAT enzyme is one of the main ways for arthropods to decompose H_2_O_2_ [31]. In the present study, the CAT activities of male and female *P. pseudoannulata* in all treatment groups were significantly higher than that of the control group. This was consistent with the changing trend of CAT for *Myzus persicae* and *H. graminicola* [29,65]. This indicated that CAT is the main pathway for the breakdown of H_2_O_2_ in *P. pseudoannulata*, so they need to maintain relatively high activity under heat stress at different times and temperatures. Unlike CAT and SOD, the GST activities of male and female *P. pseudoannulata* were not significantly different from the control group after 1 and 2 h of stress at 31 °C and 34 °C, respectively. In *Mythimna separata* and *Liposcelis bostrychophila* [28,66], GST activities significantly increased in a relatively short period of time. These changes in GST activities may result from interspecies differences. Overall, the activities of SOD, CAT, and GST all increased significantly after *P. pseudoannulata* was exposed to high temperature stress, suggested that antioxidant enzymes (SOD, CAT, and GST) play important roles in reducing oxidative damage caused by high temperature stress in *P. pseudoannulata*.

As a powerful tool for gene function studies, dsRNA-mediated silencing or inhibition of target genes has been widely used in various arthropods [67]. There were also studies using RNAi to silence in *P. pseudoannulata* [68,69]. Therefore, we performed RNAi experiments by injecting dsRNA into *P. pseudoannulata* to determine the effect of silencing HSP genes on the heat tolerance of *P. pseudoannulata*. The results showed that the expression levels of *PpHSP27*, *PpHSP60*, and *PpHSC70* genes were significantly reduced in male and female *P. pseudoannulata* after the injection of dsRNA. After silencing the *PpHSP27*, *PpHSP60*, and *PpHSC70* genes, the survival rates of female and male adult *P. pseudoannulata* were significantly decreased after high temperature stress. Therefore, our results indicated that changes in gene expression levels of *PpHSP27*, *PpHSP60* and *PpHSC70* could significantly affect the heat tolerance of spiders. Similar results have been observed for other arthropods. In *Dermatophagoides farinae*, the survival rates of *D. farinae* treated with the three ds*HSP70* fragments were significantly reduced under heat stress at 41 °C for 1 h [70]. After knockdown of *HSC70* and *HSP90* in *Plodia interpunctellalarvae*, thermotolerance was significantly reduced [71]. The survival rate of male *P. pseudoannulata* after heat stress was higher than that of female *P. pseudoannulata* after knocking down all three HSP genes. A similar phenomenon was observed in *B. tabaci*, and *D. melanogaster* [22,23]; however, the exact cause of this phenomenon is not clear at this time and further studies are needed. The study on *N. barkeri* showed that the protein level of HSP70-1 was positively correlated with the expression of *NbHSP70-1*, and the overexpression of *Nb*HSP70 protein in *Escherichia coli* (*E. coli*) enhanced the high temperature tolerance of *E. coli* [47]. Overexpression of HSP40 from *Cydia pomonell* in *E. coli* enhanced the high temperature tolerance of *E. coli* [72]. Therefore, we hypothesized that the HSC70, HSP60, HSP27 protein content of *P. pseudoannulata* correlates with the expression levels of *PpHSP27*, *PpHSP60*, and *PpHSC70* genes, and that a greater accumulation of HSC70, HSP60, and HSP27 proteins could provide strong protection against heat tolerance in *P. pseudoannulata*.

## 4. Materials and Methods

### 4.1. Spider Feeding and Experimental Handling

Adult *P. pseudoannulata* used in this study were collected in Yanlou Town, Guiyang City, Guizhou Province (106.619227° E, 26.322845° N). Before the experiment, the *P. pseudoannulata* were acclimated in an artificial climate box (25 ± 0.5 °C, relative humidity 70–80%, and photoperiod of 14 L:10 D) for two weeks and fed five housefly pupae per week. To generate heat stress, male or female *P. pseudoannulata* were placed, respectively, in a climate chamber at 31 °C, 34 °C, 37 °C, 40 °C, and 43 °C for 1, 2, 4, 8, and 12 h, and then allowed to recover for 1 h in a climate chamber at 25 °C [45]. After treatment, surviving *P. pseudoannulata* were immediately cryopreserved in liquid nitrogen and placed in a −80 °C freezer for later use. Since *P. pseudoannulata* were housed at 25 °C, 25 °C was used as a control for all treatment groups. The processed samples were divided into two parts: one for measuring antioxidant enzyme activity and the other for extracting total RNA. Each treatment consisted of three replicates (*n* = 3); 3 spiders were included in each replicate to test HSP genes expression or antioxidant enzyme activity.

### 4.2. RNA Extraction and cDNA Synthesis

The total RNA from whole *P. pseudoannulata* was extracted using a Trizol Reagent Kit (Sangon Biotech, Shanghai, China). The concentration, purity, and integrity of the RNA were determined by NanoDrop 2000 spectrophotometer (Thermo Fisher Scientific, Waltham, MA, USA) and 1% agarose gel electrophoresis. First-strand cDNA was synthesized using StarScript II RT Mix with a gDNA Remover StarScript II kit (GenStar, Beijing, China). The agarose gel electropherograms of *P. pseudoannulatais* RNA were shown in Figure S4.

### 4.3. Cloning the Full-Length cDNA of HSP Genes

Three HSP gene DNA internal fragments were amplified using the first-strand cDNA as a template. The cloning primers (Table 1) were designed using Primer Premier 6.0 (PREMIER Biosoft International, Palo Alto, CA, USA) software based on the transcriptional master sequence of the *P. pseudoannulata* [42]. The PCR reaction was performed using Taq DNA Polymerase (GenStar, Beijing, China) under the following conditions: 94 °C initial denaturation for 3 min, 35 cycles of 94 °C denaturation for 30 s, 55–60 °C annealing for 30 s, 72 °C elongation for 1–2 min, and a final 5 min extension at 72 °C. The PCR products were purified with a FastPure^®^ Gel DNA Extraction Mini Kit (Vazyme, Nanjing, China). Then, the purified products were cloned into the pGEM-T Easy vector (Promega, Madison, WI, USA) and finally transformed into *E. coli* DH5α-competent cells (Takara, Beijing, China) and sent to Sangon Biotech (Shanghai, China) for sequencing. The primers used for amplifying the 5′ and 3′ ends were designed according to the partial fragment sequences obtained by cloning and the instructions of the SMARTer^®^ RACE 5′/3′ Kit (Takara, Beijing, China). The 5′ and 3′ ends were cloned according to the instructions of the SMARTer^®^ RACE 5′/3′ Kit. The cloning steps of the 5′ and 3′ ends refer to the previous study of our task group [45]. The agarose gel electropherograms of PCR products of the clones *PpHSP27*, *PpHSP60* and *PpHSC70* are shown in Figures S5 and S6.

### 4.4. Sequence Analysis

The resulting gene fragments were assembled into the full length of each gene using the SeqMan program in DNAStar (Madison, WI, USA). AA sequences were deduced using ExPASy sequence analysis tools (https://web.expasy.org/translate/) (accessed on 18 August 2022) [73] and motifs were identified using ScanProsite software (http://www.expasy.org/tools/scanprosite) (accessed on 18 August 2022) [74]. Prediction of the relative molecular mass and theoretical isoelectric point of proteins was performed using ProtParam (http://web.expasy.org/protparam/) (accessed on 18 August 2022). The obtained AA sequences were subjected to a homology search using the NCBI BLAST program (https://blast.ncbi.nlm.nih.gov/Blast.cgi) (accessed on 18 August 2022). The MEGA-X (Mega Limited, AKL, NZ) software was used to construct a phylogenetic tree of the AA sequences of the HSPs of *P. pseudoannulata* and HSPs of other species by the neighbor-joining method, and the bootstrap value was calculated as 1000 repetitions [75].

### 4.5. Quantitative Real-Time PCR

Quantitative RT-PCR primers were designed based on the full length of the three cloned genes using primer6 software. Gene-specific primers were shown in Table 1. qRT-PCR was performed on all experimental samples using an iCycle iQ (Bio-Rad, Hercules, CA, USA). All mRNA expression levels were quantified using the *β-actin* gene as an internal reference [42]. The total volume of the qRT-PCR reaction was 20 μL and contained 10 μL of 2 × RealStar Fast SYBR qPCR Mix (GenStar, Beijing, China), 1 μL of each gene-specific primer (Table 1), 1 μL of cDNA template, and 8 μL of diethyl pyrocarbonate-treated H_2_O. The reaction conditions were as follows: 95 °C for 2 min, followed by 40 cycles of 95 °C for 15 s, 55 °C for 30 s, and 72 °C for 30 s. Melting curves were produced from 65 °C to 95 °C. Each experiment included three biological replicates and each biological replicate included three technical replicates [45]. The agarose gel electropherograms of PCR products of qPCR primers are shown in Figure S7.

### 4.6. RNA Interference

To further study the biological functions of the three HSP genes, we injected specific dsRNA into adult *P. pseudoannulata* to interfere with the expression of the three genes. The primers used for the RNAi experiments were shown in Table 1. The dsRNA of GFP was used as a negative control. Based on the manufacturer’s instructions, the dsRNA was synthesized using the Transcript Aid T7 High Yield Transcription Kit (Thermo Fisher Scientific, Waltham, MA, USA). The agarose gel electropherograms of dsRNA are shown in Figure S8.

A total of 99 male and 99 female *P. pseudoannulata* were used for each HSP gene interference experiment. dsRNA injections were performed using a microsyringe (HAMILTON, Shanghai, China). The spider was immobilized by hand (wearing gloves to prevent being bitten by the spider) and dsRNA was slowly injected from the back of the spider’s abdomen, with 1000 ng/500 nL of dsRNA injected into each spider. The injected *P. pseudoannulata* were reared in an artificial climate box (25 ± 0.5 °C, relative humidity 70–80%, and photoperiod of 14 L:10 D) and after 72 h post-injection of dsRNA, 9 female and 9 male *P. pseudoannulata* from each treatment group were randomly collected, frozen in liquid nitrogen, and stored in a −80 °C freezer for subsequent verification of RNAi efficiency. Each treatment consisted of three replicates (*n* = 3); 3 spiders were included in each replicate to determine silencing efficiency. The remaining 90 male and 90 female *P. pseudoannulata* were used to verify the effect of each HSP gene on spider thermotolerance. The injected male and female *P. pseudoannulata* were placed in a climate chamber at 43 °C for 4 h and survival rate was recorded. Each treatment consisted of three replicates (*n* = 3), 30 spiders were included in each replicate for the survival rate determination. 

### 4.7. Antioxidant Enzyme Activity Detection

SOD, CAT, and GST activities were determined using commercial kits (Cat. No.: BC0175, BC0200, BC0350, Solarbio, Beijing, China). The three *P. pseudoannulata* were mixed with the 0.05 M pH 7.0 phosphate buffers and homogenized in an ice bath (adding 1 mL of buffer for every 0.1 g of spider), then 1 mL of the product after homogenization in ice bath was centrifuged at 12,000× *g* for 10 min, and the supernatant was put on ice for use. The absorbance was read in a Multiskan FC (Thermo Fisher Scientific, Waltham, MA, USA) with reference to the kit instructions and the antioxidant enzyme activity was calculated according to the fresh weight (FW) of the sample [76].

### 4.8. Data Analysis

According to the formula provided by the kit, SOD, CAT, and GST activities were calculated. The relative expression of the HSP gene for each treatment was calculated by the 2^−ΔΔCt^ method [77]. All data analysis in this study was performed using SPSS 22 software (SPSS Inc., Chicago, IL, USA). In the data analysis of HSP gene expression and antioxidant enzyme activity of *P. pseudoannulata* under different conditions of high temperature stress, separate one-way Analysis of Variance (ANOVA) were used for each group of temperature or gender, followed by pairwise comparisons at different time points using Tukey’s Honest Significant Difference (HSD) method (*P* = 0.05). In the verification experiment of silence efficiency, the Student’s t-test was used to compare the difference of *PpHSP* gene expression in *P. pseudoannulata* after being injected with ds*GFP* and ds*HSP* (*P* = 0.05). In the function verification experiment of *PpHSP* genes, separate one-way ANOVAs were used for two genders, Tukey’s HSD method was used, followed by pairwise comparisons with different RNAi processing (*P* = 0.05), and the Student’s t-test was used to compare the difference in the survival rate of female and male *P. pseudoannulata* after the HSP genes were silenced (*P* = 0.05). All the data are expressed as the mean ± standard deviation (SD).

## 5. Conclusions

We cloned the full length of *PpHSP27*, *PpHSP60*, and *PpHSC70* of *P. pseudoannulata* and analyzed their sequence features. Trends in the expression of these HSP genes and the enzymatic activities of SOD, CAT, and GST were explored at different high temperatures and stress durations, which revealed that they were significantly upregulated with increasing temperature and stress durations. We successfully knocked down HSP genes and verified their functions in *P. pseudoannulata*. The results indicated that knockdown of *PpHSP27*, *PpHSP60*, and *PpHSC70* enhanced susceptibility to high temperatures in *P. pseudoannulata*. In conclusion, our study shows that SOD, CAT, and GST enable *P. pseudoannulata* to remove the metabolic byproducts produced by heat stress. *PpHSP27*, *PpHSP60*, and *PpHSC70* are key factors that affect the heat tolerance of *P. pseudoannulata*. They all play an important role in the resistance of *P. pseudoannulata* to high temperature.

## Figures and Tables

**Table 1:** Primer names and sequences used in this study.

Primer Names	Primer Sequences	Use of Primers
*PpHSP27*-1F	GGTTCCAGAAGCGTCTCT	Amplification of internal HSP genes fragment
*PpHSP27*-1R	CGTCCGAGTCCTGTATGAG	
*PpHSP27*-2F	CATTCAGAGCCACAGCACAG	
*PpHSP27*-2R	TTCAGAAGCGGTTACAGAGAAC	
*PpHSP60*-F	GGTGTAGATGTCTTGGCTGAT	
*PpHSP60*-R	CAGTTGGAATGGCATGAGTTC	
*PpHSC70*-F	ATGTGTTGCTGTTATGGATGG	
*PpHSC70*-R	ATGCTTGCTGTAGTTCTTGTG	
3′-*PpHSP27*-F	CAGATGGCGTCCTCACAGT	3′RACE
3′-*PpHSP60*-F	GATGCTGCTGTAGTCACTCA	
3′-*PpHSC70*-F	AGAGTCACCAGAAACATTGCGTAAAG	
5′-*PpHSP27*-R	TATGAGGGGCGAGCTGAGTCCGTCGAGC	5′RACE
5′-*PpHSP60*-R	GCCTCCAACCTTGAGAAGTGCAACACCA	
5′-*PpHSC70*-R	CCCTTGAGCTTCAACCCAAGCATCACC	
q*PpHSP27*-F	GGTTCAAGCCAAACACGAAGA	qPCR
q*PpHSP27*-R	GTGAGGACGCCATCTGTGA	
q*PpHSP60*-F	TGATGCTGCTGTAGTCACTCA	
q*PpHSP60*-R	ACCCATACCGCCCATACCT	
q*PpHSC70*-F	GGCTATATCTGATGCTGATGTGA	
q*PpHSC70*-R	AAGAACACCACCCTGAATTGC	
*β-actin*-F	GACCCAATACTTCTAACG	
*β-actin*-R	ACAGCAGGAAACACTTA	
ds*PpHSP27*-F	taatacgactcactatagggTGAGGAGTGACTTCAGCGTG	RNAi
ds*PpHSP27*-R	taatacgactcactatagggCAGGCTGATATTGGCTGACA	
ds*PpHSP60*-F	taatacgactcactatagggCTCCAGAAGAAATAGCCCAGGTT	
ds*PpHSP60*-R	taatacgactcactatagggAACGAGATCAGCATCATCACCAA	
ds*PpHSC70*-F	taatacgactcactatagggGCTGGTGATGTCACAGATGTT	
ds*PpHSC70*-R	taatacgactcactatagggCTTCTTCAGGTAACTGGCTCTT	
ds*GFP*-F	taatacgactcactatagggGCCAACACTTGTCACTACTT	
ds*GFP*-R	taatacgactcactatagggGGAGTATTTTGTTGATAATGGTC	

The lowercase letters in the primer sequences represent the sequence of the T7 promoter.

## Data Availability

The data and materials supporting the conclusions of this study are included within the article.

## Supplementary Materials

The following supporting information can be downloaded at: https://www.mdpi.com/article/10.3390/ijms232112821/s1.
