# Supplementary material for: Three Heat Shock Protein Genes and Antioxidant Enzymes Protect Pardosa pseudoannulata (Araneae: Lycosidae) from High Temperature Stress
Source: Int J Mol Sci. 2022 Oct 24;23(21):12821. doi: 10.3390/ijms232112821 (PMC9655195; doi:10.3390/ijms232112821)
Supplement: Supplementary file 1 [file ijms-23-12821-s001.zip › ijms-1932741-supplementary.pdf]

```
1     CATATTTCATTTCATTTCAACCCGGTTCCAGAAGCGTCTCTGGCGTCTGTTTCAAACTCCTTTGGTTTATTCCTCTTCTCTGAATTCCAA
91    AACATCTAAAAACCAGCCATGTCTTTCGAGCGCCGCCTTCCCCTCTTGAGGAGTGACTTCAGCGTGATTGACCGCGAGTTCAGCTCGATC
1                         M  S  F  E  R  R  L  P  L  L  R  S  D  F  S  V  I  D  R  E  F  S  S  I
181   CGCGAAAAGTTTGACCAGGAGATGCGTCTGATGGAGGAGGAGATGAACCGCTTCAGGTCTCAACTGTTGGACCGTGAGGTGCCCCGCATT
25     R  E  K  F  D  Q  E  M  R  L  M  E  E  E  M  N  R  F  R  S  Q  L  L  D  R  E  V  P  R  I
271   CAGAGCCACAGCACAGCCACCGGCACCGACGGACAGACCACACACAGGTCCGAGGTGAGGACGTGGCTCGACGGACTCAGCTCGCCCCTC
55     Q  S  H  S  T  A  T  G  T  D  G  Q  T  T  H  R  S  E  V  R  T  W  L  D  G  L  S  S  P  L
361   ATACAGGACTCGGACGACGGAAAGAAGCTCAGGCTCAGGTTCGATGTCAGCCAATATCAGCCTGAGGAGATCGTCGTCAAGACAGTAGAC
85     I  Q  D  S  D  D  G  K  K  L  R  L  R  F  D  V  S  Q  Y  Q  P  E  E  I  V  V  K  T  V  D
451   AACAAACTACAGGTTCAAGCCAAACACGAAGAAAAATCTGACACTAAGTCTGTGTACCGCGAGTACAACAGAGAATTCCTCCTTCCCAAG
115    N  K  L  Q  V  Q  A  K  H  E  E  K  S  D  T  K  S  V  Y  R  E  Y  N  R  E  F  L  L  P  K
541   GGAACAAATCCTGAAATGATCAAATCCTCTCTGTCAACAGATGGCGTCCTCACAGTCGAAGCTCCTCTACCACCAGCTTTAGAAGCAGGC
145    G  T  N  P  E  M  I  K  S  S  L  S  T  D  G  V  L  T  V  E  A  P  L  P  P  A  L  E  A  G
631   GTTCATATGATACCCATCGAAAACAAATAAACTTTTTAAAAAATCCATTTTTATATCGTTTACAGAAACTACAAGTTGCTAAAAAATTCG
175    V  H  M  I  P  I  E  N  K  *
721   AGTTGTACTGGAAACACATAGTATTTCTTGTAGTTTTATATAAAAATAAAAAAATATTGATGTATTTATCATGGATGAAAATGTTCGACT
811   CATGGATATTTCACGGTATTGGTTCTCTGTAACCGCTTCTGAAATTTTTTTTATTACAAGTCACCTAATGTCATTAATTTCATTCTTTA
901   TTTCTGTGCCGTAAATTTTCTTGTGATGATGTTAAATTAATGCAGTTCGGCAAATAAAATTACATTCTGAAAAAAAAAAAAAAAAAAAAA
991   AAAAAAA
```

Figure S1. Nucleotide and deduced AA sequences of *PpHSP27* from *P. pseudoannulatais*. A putative polyadenylation signal is marked in blue. Characteristic sequences of the small heat shock proteins (sHSP) family are marked in yellow. Promoters and terminators are marked in green.

```
1       GTTACAATTTTCGGCTCCTGAGCCGCACATGCTTTTTGATAATTTACTATAGGGGCTCTCTGCTTCAGTATTGACACTTCTTACTCCTTT
91      TGTAAATTTGAGTCTAATTTATTTTCTCGCTTTATATTTACTTCTATAGGTATAGTTTTAAAGTCATCATGTACAGATTACACCATTTAA
1                                                                               M  Y  R  L  H  H  L
181     CCCGACCTCTCTCTCGACAAATATCATGGAGTATTAGAAGAAATTATGCTAAAGATGTCAAATTTGGCCCAGATGTTAGAGCAGCTATGC
8       T  R  P  L  S  R  Q  I  S  W  S  I  R  R  N  Y  A  K  D  V  K  F  G  P  D  V  R  A  A  M
271     TACAAGGTGTAGATGTCTTGGCTGATGCAGTTGCTGTAACAATGGGACCAAAGGGTAGAAATGTCATTATTGAACAGTCCTGGGGCTCTC
38      L  Q  G  V  D  V  L  A  D  A  V  A  V  T  M  G  P  K  G  R  N  V  I  I  E  Q  S  W  G  S
361     CCAAAATTACAAAAGATGGTGTTACTGTTGCAAAAGCTATTGAACTTAAGGATAAGTTCCAAAATATTGGAGCTAAACTTGTTCAAGATG
68      P  K  I  T  K  D  G  V  T  V  A  K  A  I  E  L  K  D  K  F  Q  N  I  G  A  K  L  V  Q  D
451     TTGCAAATAATACTAATGAAGAAGCAGGAGATGGTACAACAACAGCTACAGTATTAGCAAGAGCAATTGCTAAAGAAGGATTTGAAAAAA
98      V  A  N  N  T  N  E  E  A  G  D  G  T  T  T  A  T  V  L  A  R  A  I  A  K  E  G  F  E  K
541     TTAGCAAAGGAGCTAACCCAATTGAATTTAGGAAAGGTGTCATGCTAGCTGTTGAAAGAGTTGTTGAAGAATTAAGAAAAAATTCAAAAC
128     I  S  K  G  A  N  P  I  E  F  R  K  G  V  M  L  A  V  E  R  V  V  E  E  L  R  K  N  S  K
631     CTGTTACAACTCCAGAAGAAATAGCCCAGGTTGCTACAATTTCTGCAAATGGAGATAAAACAATAGGTGATCTTATATCTAGTGCTATGA
158     P  V  T  T  P  E  E  I  A  Q  V  A  T  I  S  A  N  G  D  K  T  I  G  D  L  I  S  S  A  M
721     AAAAAGTTGGAAAAGATGGTGTAATTACTGTCAAAGATGGAAAGACATTGAGTGACGAATTAGAAGTTATTGAAGGCATGAAATTTGATA
188     K  K  V  G  K  D  G  V  I  T  V  K  D  G  K  T  L  S  D  E  L  E  V  I  E  G  M  K  F  D
811     GGGGTTATATATCACCCTATTTCATCAATACAGCTAAAGGTGCCAAAGTTGAATATCAAGATGCTCTAGTTTTGTTTAGTGAAAAGAAAA
218     R  G  Y  I  S  P  Y  F  I  N  T  A  K  G  A  K  V  E  Y  Q  D  A  L  V  L  F  S  E  K  K
901     TATCTAGTGTACAGTCAATTATACCTGCACTTGAATTAGCTAACTCAAAGCGAAAGCCTCTTATAATTGTTGCTGAAGATGTAGATGGTG
248     I  S  S  V  Q  S  I  I  P  A  L  E  L  A  N  S  K  R  K  P  L  I  I  V  A  E  D  V  D  G
991     AAGCTTTAAGTACTATGGTTCTGAACAGGTTAAAGGTTGGTTTGCAAGTAGCTGCAGTCAAAGCTCCTGGTTTTGGAGATAACAGAAAAA
278     E  A  L  S  T  M  V  L  N  R  L  K  V  G  L  Q  V  A  A  V  K  A  P  G  F  G  D  N  R  K
1081    GTACTCTTCATGATATGGCCATTGCTACAGGTGGTATTGTTTTTGGTGATGATGCTGATCTCGTTAAACTAGAGGATGTTCAGCCAACAG
308     S  T  L  H  D  M  A  I  A  T  G  G  I  V  F  G  D  D  A  D  L  V  K  L  E  D  V  Q  P  T
1171    ATCTCGGAGAGGTTTCTGAAGTTGTTATTACCAAAGATGATACTCTATTGCTTAAGGGAAAGGGCAACAAGCAAGACATTGATAGAAGAA
348     D  L  G  E  V  S  E  V  V  I  T  K  D  D  T  L  L  L  K  G  K  G  N  K  Q  D  I  D  R  R
1261    TTGCTCAAATCAAAGATGAGATTGAACAAACAACGTCCCAGTATGAGAAAGAAAAACTTCAAGAAAGACTTGCAAGGCTATCTAATGGTG
378     I  A  Q  I  K  D  E  I  E  Q  T  T  S  Q  Y  E  K  E  K  L  Q  E  R  L  A  R  L  S  N  G
1351    TTGCACTTCTCAAGGTTGGAGGCTCTAGTGAAGTAGAAGTAAATGAGAAAAAGGACAGAGTGACTGATGCATTGAATGCAACCCGTGCTG
408     V  A  L  L  K  V  G  G  S  S  E  V  E  V  N  E  K  K  D  R  V  T  D  A  L  N  A  T  R  A
1441    CGGTTGAAGAAGGAATTGTTGCTGGTGGAGGAACAGCCCTTTTAAGATGCTTACCAGCTCTTGATGAAATTTCTTATGAAAATGAAGACC
438     A  V  E  E  G  I  V  A  G  G  G  T  A  L  L  R  C  L  P  A  L  D  E  I  S  Y  E  N  E  D
1531    AGAAAATTGGTGTTGACATAGTCAGGCGATCTCTGAAGATGCCATGCACACAAATTGCAATGAATGCTGGTGTTGATGCTGCTGTAGTCA
468     Q  K  I  G  V  D  I  V  R  R  S  L  K  M  P  C  T  Q  I  A  M  N  A  G  V  D  A  A  V  V
1621    CTCAAAAAGTTCTGGACAACAAAGGTGAATTTGGTTATGATGCATTGCGGGGAGAATATGTAAACATGATTGAAGCTGGAATAATTGATC
498     T  Q  K  V  L  D  N  K  G  E  F  G  Y  D  A  L  R  G  E  Y  V  N  M  I  E  A  G  I  I  D
1711    CTACAAAAGTGGTAAGGACAGCTTTAATGGATGCTGCTGGTGTTGCATCTCTTCTTACTACTGCTGAATCTGTTATTGTTGAAATCCCCA
528     P  T  K  V  V  R  T  A  L  M  D  A  A  G  V  A  S  L  L  T  T  A  E  S  V  I  V  E  I  P
1801    AAGAAGAGAAACCTCCAATGGGTGGTGGCATGGGAGGTATGGGCGGTATGGGTGACTATGGAATGTAACAACCTAATCAAATTAGATAGT
558     K  E  E  K  P  P  M  G  G  G  M  G  G  M  G  G  M  G  D  Y  G  M  *
1891    TTTGTGCTGAACTTTTAAAATATCAGGAACACTGCAGGACATTACAGTGGCAACATTATGCATTTGAACTCATGCCATTCCAACTGCATC
1981    ATGTCTCACATTGTTTGTTAATTCATTTGTAGAGTCTTATATAATAAATTTTCATAAAAAAAAAAAAAAAAAAAAAAAAAAAAA
```

Figure S2. Nucleotide and deduced AA sequences of *PpHSP60* from *P. pseudoannulatais*. A putative polyadenylation signal is marked in blue. Characteristic sequences of the HSP60 family are marked in yellow. Promoters and terminators are marked in green.

```
1     GCATGTGCTATTTCGTCAGACCTTTTACCTTCAGCAATAGTGCTTTGTCCTTGTGCGGTTAAAAGCCGAGTTAAATCGCTTTTCTCGTGT
91    AATTCCTATTTTGTATAGTTCAAGAATTTTTTATATAAACAAAATGCTTAAAATTTTAAGAAATACGAATTCAAATTGTCTTAGATCATC
1                                                  M  L  K  I  L  R  N  T  N  S  N  C  L  R  S  S
181   CGTTTTAGGCAGTTTCTATGGTTCAAGATTCCTCTCACATCAAGCACATAATAAGGCACTGCTTTATGTTGCCAACAAAAATGGACTTCA
17     V  L  G  S  F  Y  G  S  R  F  L  S  H  Q  A  H  N  K  A  L  L  Y  V  A  N  K  N  G  L  Q
271   AATGCGATATAAGTCTGATGCTGTTAAAGGAAGTGTCATTGGCATTGATTTGGGAACAACGAACTCATGTGTTGCTGTTATGGATGGCAA
47     M  R  Y  K  S  D  A  V  K  G  S  V  I  G  I  D  L  G  T  T  N  S  C  V  A  V  M  D  G  K
361   AACTCCAAAAGTAATAGAAAATTCTGAAGGTTCAAGGACTACACCTTCTGTTGTTGCAATCACAAAAGATGGTGAAAGATTAGTTGGAAT
77     T  P  K  V  I  E  N  S  E  G  S  R  T  T  P  S  V  V  A  I  T  K  D  G  E  R  L  V  G  M
451   GCCAGCTAAAAGACAAGCTATTACCAATGCAGCTAACACACTTTCTGCCACAAAGCGTTTGATCGGTCGTAGGTTTGAGGATCCTGAGGT
107    P  A  K  R  Q  A  I  T  N  A  A  N  T  L  S  A  T  K  R  L  I  G  R  R  F  E  D  P  E  V
541   GCAAAAAGACCAGAAAACAGCCTCATTCAAAATAGTTAAAGCATCAAATGGTGATGCTTGGGTTGAAGCTCAAGGGAAAATGTATTCTCC
147    Q  K  D  Q  K  T  A  S  F  K  I  V  K  A  S  N  G  D  A  W  V  E  A  Q  G  K  M  Y  S  P
631   AAGCCAAATTGGTGCTTTTATTTTAATGAAAATGAAAGAGACAGCAGAGAGTTGTTTAGGACAAACTGTTAAAAATGCTGTCATTACTGT
177    S  Q  I  G  A  F  I  L  M  K  M  K  E  T  A  E  S  C  L  G  Q  T  V  K  N  A  V  I  T  V
721   TCCTGCATATTTCAATGATTCTCAACGACAGGCAACCAAAGATGCAGGCCAGATTGCAGGACTTAATGTACTTCGTGTAATTAATGAACC
207    P  A  Y  F  N  D  S  Q  R  Q  A  T  K  D  A  G  Q  I  A  G  L  N  V  L  R  V  I  N  E  P
811   TACTGCTGCAGCCTTAGCTTATGGTATTGATAAAACAGATAAAATAATTGCTGTTTATGATTTGGGTGGGGGTACATTTGATATATCAGT
247    T  A  A  A  L  A  Y  G  I  D  K  T  D  K  I  I  A  V  Y  D  L  G  G  G  T  F  D  I  S  V
901   TCTTGAAATTCAGAAAGGTGTTTTTGAAGTAAAATCTACTAATGGAGATACTTTCTTGGGTGGTGAAGATTTTGACAATACTCTTGTTAA
277    L  E  I  Q  K  G  V  F  E  V  K  S  T  N  G  D  T  F  L  G  G  E  D  F  D  N  T  L  V  N
991   TTATTTGGCAAAGGAGTTTAAAAAAGAACAAGGAGTTGATTTATCAAAAGACACTATGGCAATGCAAAGATTAAAAGAAGCAGCTGAAAA
307    Y  L  A  K  E  F  K  K  E  Q  G  V  D  L  S  K  D  T  M  A  M  Q  R  L  K  E  A  A  E  K
1081  AGCTAAAATAGAGTTATCTTCATCTGTGCAAACAGATATTAACTTGCCATATCTGACTGTTGATGCAAGTGGACCAAAGCATTTGAATAT
337    A  K  I  E  L  S  S  S  V  Q  T  D  I  N  L  P  Y  L  T  V  D  A  S  G  P  K  H  L  N  I
1171  AAAATTATCTAGAGCTAAGCTTGAGAGTTTGGTGGCTGACCTAATCAAAAGGACAATTGAACCTTGCAAAAAGGCTATATCTGATGCTGA
367    K  L  S  R  A  K  L  E  S  L  V  A  D  L  I  K  R  T  I  E  P  C  K  K  A  I  S  D  A  D
1261  TGTGAAAAAAACTGATATTCAAGAAGTAATTTTAGTTGGTGGAATGACTAGAATGCCAAAGGTACAAGAAGTTGTACAAGAAATTTTTGG
397    V  K  K  T  D  I  Q  E  V  I  L  V  G  G  M  T  R  M  P  K  V  Q  E  V  V  Q  E  I  F  G
1351  AAGAGTCCCTAGTAAATCTGTGAATCCTGATGAAGCTGTTGCTGTTGGAGCTGCAATTCAGGGTGGTGTTCTTGCTGGTGATGTCACAGA
427    R  V  P  S  K  S  V  N  P  D  E  A  V  A  V  G  A  A  I  Q  G  G  V  L  A  G  D  V  T  D
1441  TGTTTTACTTTTAGATGTTACTCCTTTGTCATTGGGTATTGAAACTCTTGGTGGCGTTTTCACAAAACTTATTACAAAAAATACAACCAT
457    V  L  L  L  D  V  T  P  L  S  L  G  I  E  T  L  G  G  V  F  T  K  L  I  T  K  N  T  T  I
1531  TCCAACCAAAAAGAGTCAGATATTTTCTACTGCAGCAGATGGGCAGACACAAGTAGAAATTAAAGTATTCCAAGGAGAAAGAGAAATGGC
487    P  T  K  K  S  Q  I  F  S  T  A  A  D  G  Q  T  Q  V  E  I  K  V  F  Q  G  E  R  E  M  A
1621  TGCAGATAACAAACTTTTAGGACAGTTTTCATTGGTTGGCATTCCACCTGCACCCAGGGGTGTTCCTCAAATTGAGGTTACATTTGATAT
517    A  D  N  K  L  L  G  Q  F  S  L  V  G  I  P  P  A  P  R  G  V  P  Q  I  E  V  T  F  D  I
1711  TGATGCTAATGGAATTGTCCATGTCTCTGCCCGAGACAGAGCTACTGGCAAAGAACAAAACATTGTCATTCAGTCATCTGGTGGTTTAAG
547    D  A  N  G  I  V  H  V  S  A  R  D  R  A  T  G  K  E  Q  N  I  V  I  Q  S  S  G  G  L  S
1801  TAAAGATGAAATTGAAAATATGATTAAGAATGCTGAGAAATATGCTGAACAAGATAAAATTAAAAAGGAAACAGTTGAAGTTGTCAACCA
577    K  D  E  I  E  N  M  I  K  N  A  E  K  Y  A  E  Q  D  K  I  K  K  E  T  V  E  V  V  N  Q
1891  AGCAGAAGGTATAATTCATGATACGGAATCTAAGATGGAAGAGTTTAAGAGCCAGTTACCTGAAGAAGAGGTTACAAACATCAAAAACAA
607    A  E  G  I  I  H  D  T  E  S  K  M  E  E  F  K  S  Q  L  P  E  E  E  V  T  N  I  K  N  K
1981  AATTAATGAAGTTAGGCAATTAATTACAAACAAAGATCAAGAGTCACCAGAAACATTGCGTAAAGCAACACAAGAACTACAGCAAGCATC
637    I  N  E  V  R  Q  L  I  T  N  K  D  Q  E  S  P  E  T  L  R  K  A  T  Q  E  L  Q  Q  A  S
2071  TCTGAAGTTGTTTGAAGCTGCTTACAAAAAGATGGCTAATGAAAGGCAAGGTGGTTCAACAGAAAGCAGTAGCAGTGAAAGTTCAAGTAC
667    L  K  L  F  E  A  A  Y  K  K  M  A  N  E  R  Q  G  G  S  T  E  S  S  S  S  E  S  S  S  T
2161  TGAAGAAGCCGAAAAGAAAGAAGAAAAAAATTAAATAAGAAATCATGAAATTTTATGTGTGGGACTGATTCCAATAGCTGGCTATATATA
697    E  E  A  E  K  K  E  E  K  N  *
2251  TATTGCCATTTTGCCTGTGCTGTGCATTAAGAAACGTCATTGTAATATTTAAGTAATCAAAAGATGTTGATTACTGCAGAACCACTTGCA
2341  TTGTATTATAGCTCATCATTAACTCTGTAGATAAAAGATGTCTAAAAAATTAATAATCTTTTATTTCATATGAAAAAAAAAAAAAAAAAA
2431  AAAAAAAA
```

Figure S3. Nucleotide and deduced AA sequences of *PpHSC70* from *P. pseudoannulatais*. A putative polyadenylation signal is marked in blue. Characteristic sequences of the HSP70 family are marked in yellow, violet, and red. Promoters and terminators are marked in green.

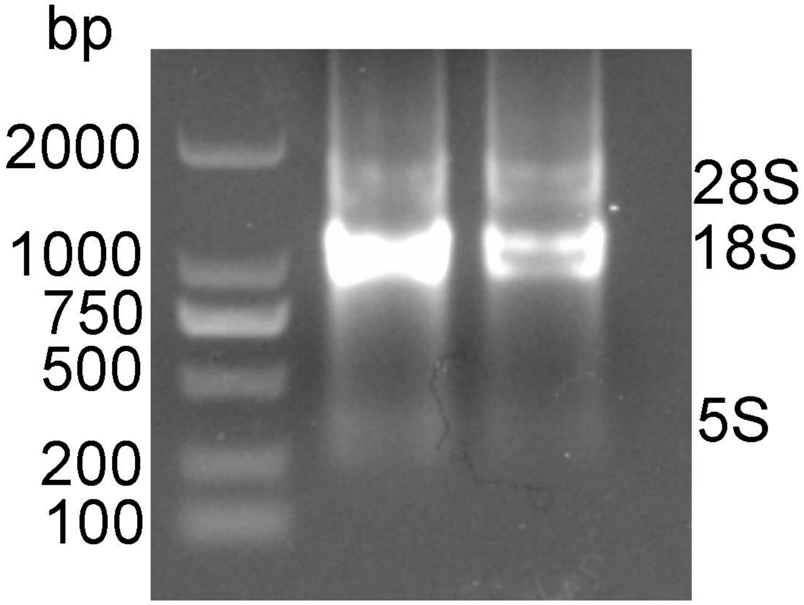

Figure S4. Agarose gel electropherogram of *P. pseudoannulatais* RNA.

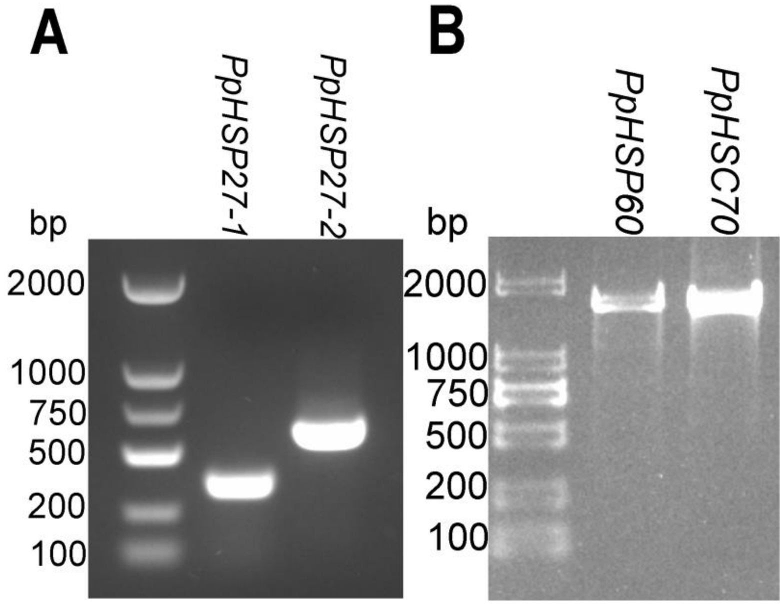

Figure S5. Agarose gel electropherogram of *PpHSP27*, *PpHSP60*, and *PpHSC70* intermediate fragments polymerase chain reaction (PCR) products. (A) *PpHSP27*, (B) *PpHSP60*, and *PpHSC70*.

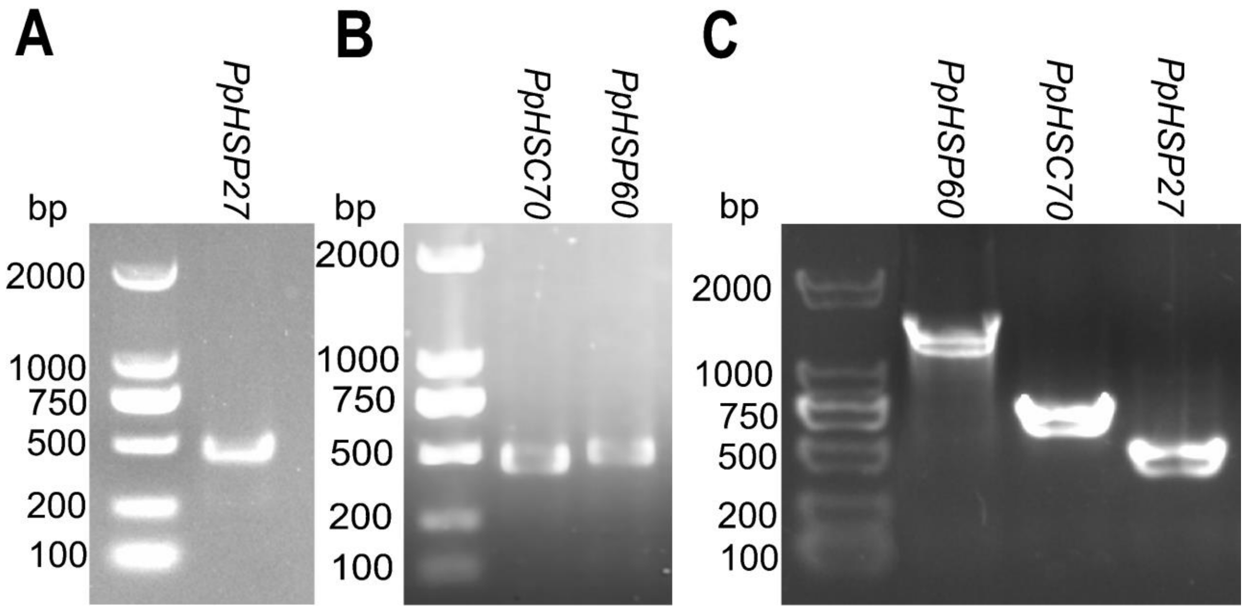

Figure S6. Agarose gel electropherogram of *PpHSP27*, *PpHSP60*, and *PpHSC70* untranslated region (UTR) PCR products. (A) *PpHSP27* 3′(UTR), (B) *PpHSP60*, and *PpHSC70* 3′ UTR. (C) *PpHSP27*, *PpHSP60*, and *PpHSC70* 5′ UTR.

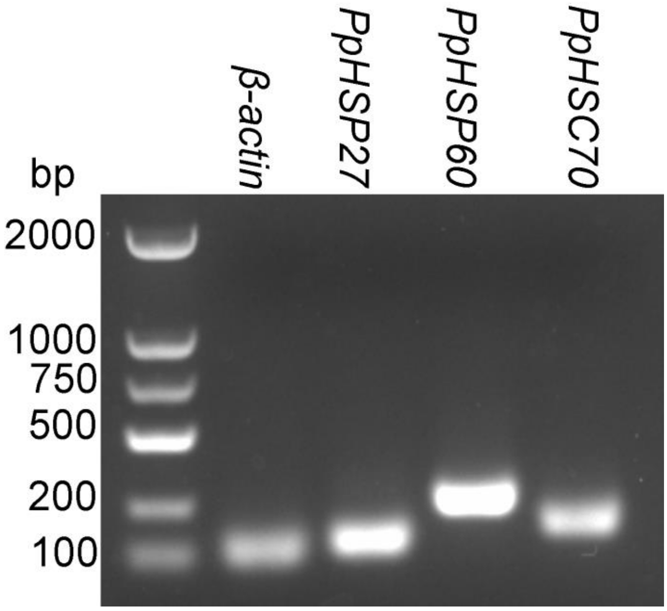

Figure S7. Agarose gel electropherogram of PCR products of qPCR primers.

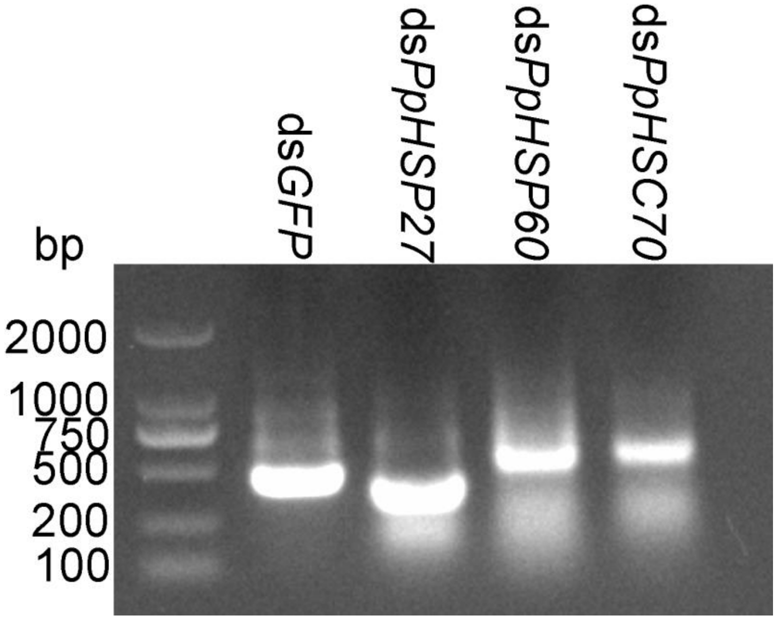

Figure S8. Agarose gel electropherogram of dsRNA.
